# Calcium dobesilate reduces VEGF signaling by interfering with heparan sulfate binding site and protects from vascular complications in diabetic mice

**DOI:** 10.1371/journal.pone.0218494

**Published:** 2020-01-14

**Authors:** Florence Njau, Nelli Shushakova, Heiko Schenk, Vera Christine Wulfmeyer, Robin Bollin, Jan Menne, Hermann Haller

**Affiliations:** Division of Nephrology, Hannover Medical School, Hannover, Germany; Medical College of Wisconsin, UNITED STATES

## Abstract

Inhibiting vascular endothelial growth factor (VEGF) is a therapeutic option in diabetic microangiopathy. However, VEGF is needed at physiological concentrations to maintain glomerular integrity; complete VEGF blockade has deleterious effects on glomerular structure and function. Anti-VEGF therapy in diabetes raises the challenge of reducing VEGF-induced pathology without accelerating endothelial cell injury. Heparan sulfate (HS) act as a co-receptor for VEGF. Calcium dobesilate (CaD) is a small molecule with vasoprotective properties that has been used for the treatment of diabetic microangiopathy. Preliminary evidence suggests that CaD interferes with HS binding sites of fibroblast growth factor. We therefore tested the hypotheses that (1) CaD inhibits VEGF signaling in endothelial cells, (2) that this effect is mediated via interference between CaD and HS, and (3) that CaD ameliorates diabetic nephropathy in a streptozotocin-induced diabetic mouse model by VEGF inhibition. We found that CaD significantly inhibited VEGF_165_-induced endothelial cell migration, proliferation, and permeability. CaD significantly inhibited VEGF_165_-induced phosphorylation of VEGFR-2 and suppressed the activity of VEGFR-2 mediated signaling cascades. The effects of CaD in vitro were abrogated by heparin, suggesting the involvement of heparin-like domain in the interaction with CaD. In addition, VEGF_121_, an isoform which does not bind to heparin, was not inhibited by CaD. Using the proximity ligation approach, we detected inhibition of interaction in situ between HS and VEGF and between VEGF and VEGFR-2. Moreover, CaD reduced VEGF signaling in mice diabetic kidneys and ameliorated diabetic nephropathy and neuropathy, suggesting CaD as a VEGF inhibitor without the negative effects of complete VEGF blockade and therefore could be useful as a strategy in treating diabetic nephropathy.

## Introduction

Diabetic nephropathy is one of the most important microvascular complications of diabetes mellitus and is responsible for 40–50% of all cases of end-stage renal disease (ESRD), despite various treatment strategies, such as intensive blood glucose control [1,2], lowering of blood pressure [3,4] or renin-angiotensin-system blockade [5] that have been established over the last 20 years [6,7]. The complex pathogenesis of diabetic nephropathy makes the development of evidence-based therapeutic strategies difficult [8].

An increased expression of vascular endothelial growth factor (VEGF) has been observed in rat and mice models of diabetes and in diabetic patients [9–12]. Increased VEGF-A/VEGFR-2 signaling contributes to renal disease in several important ways, including vascular permeability [13], vasodilation, hyperfiltration [14,15], capillary growth, and monocyte chemotaxis [16,17]. Inhibiting VEGF seems to prevent the development of nephropathy in animal models. Treatment with an anti-VEGF_165_ antibody results in a significant attenuation of albuminuria in diabetic mice and rats[1,14,18]. However, anti-VEGF treatment in the prevention of microvascular disease is associated with serious obstacles, since, for example, VEGF_165_ antibodies cause renal damage and hypertension in lung cancer patients, and nephrotoxicity commonly occurs after anti-VEGF therapy as previously reviewed [19,20]. VEGF has been observed to have an important role in maintaining the endothelial integrity because, anti-VEGF therapy in patients with solid tumours as well as conditional ablation of VEGF in adult mice led to microangiopathy [21,22]. These conflicting observations have led to the hypotheses that, under physiological conditions VEGF signaling is necessary to maintain endothelial stability, however, overexpressing VEGF and its signaling, as it is observed in diabetes, leads to endothelial damage and microvascular diseases.

Calcium dobesilate (CaD) is a small molecule which has been used in particular in Asia and South America to treat various vascular disorders including diabetic microvascular disease, for years. At present, CaD is approved in numerous countries for the treatment of diabetic retinopathy another important complication of diabetes mellitus and its efficacy has been analyzed in a recent meta-analysis [23,24]. Moreover, recent studies have demonstrated that CaD can be safely and effectively used to treat diabetic nephropathy in type 2 diabetic patients [25,26]. However, the pharmacology of CaD is poorly understood. CaD belongs to the 2,5-dihydroxyphenylic acids, a newly described family of molecules which interfere with growth factor signaling [27,28], CaD binds to the heparin-binding domain of Fibroblast Growth Factor-1 (FGF-1), thus reducing FGF-1 activity [27]. We reasoned that CaD could function as a novel VEGF antagonist. We used cultured endothelial cells and animal models and found that CaD indeed reduces exaggerated VEGF signaling, while maintaining physiological effects of VEGF. The 2,5-dihydroxyphenylic-acid compound class could represent a novel VEGF antagonist without adverse side effects.

## Materials and methods

### Materials

Primary human umbilical vein endothelial cells (HUVECs; ATCC^®^PCS-100.010) were purchased from ATCC (Wesel, Germany) and cultured in EGM^™^ BulletKit^™^ without exogenous VEGF (Lonza). CCK-8 cell viability assay kit was purchased fromDojindo Molecular Technologies, Munich Germany and polycarbonate filters (ThinCert^™^) was from Greiner bio-one. All VEGF-A used in this study were VEGF_165_ isoform unless designated otherwise. The recombinant VEGF_165_, VEGF_121_ and biotinylated-VEGF_165_ (bt-VEGF_165_), VEGFR-1, VEGFR-2 and recombinant Human Active Heparanase (HPSE; 7570-GH) were from R&D Systems Inc. (Wiesbaden-Nordenstadt, Germany). Heparin sodium salt from porcine intestinal mucosa and Calcium dobesilate (2,5-Dihydroxybenzenesulfonic acid calcium salt), Fluorescein isothiocyanate-dextran, molecular mass: 70 kDa, Duolink^®^ In Situ PLA kit and probes, calcein-AM and streptozotozin (STZ) were from Sigma Aldrich (Taufkirchen, Germany). Rabbit primary antibodies for VEGFR-2, p-Tyr1175, p-ERK1/2, ERK1/2, p-P38, p-MEK and MEK were acquired from Cell Signaling Technology (Leiden, The Netherlands) and F4/80 (clone A3-1; BioLegend, San Diego, CA, USA). Mouse anti-heparan sulfate proteoglycan (mAb F58-10E4) was from Amsbio. GAPDH and all secondary antibodies (except Cy3, Jackson ImmunoResearch, West Grove, USA) were from Santa Cruz Biotechnology (Heidelberg, Germany). Streptavidin-HRP, Alexa fluor 488 (Thermo Fisher Scientific) and phalloidin-Alexa fluor 488 was from Invitrogen, Carlsbad, CA, USA. Other primary antibodies were obtained as indicated; Occludin (Invitrogen), Claudin-5 (Bioworld Technology, Inc), ZO-1 (BD Transduction Laboratories), Vinculin (Chemicon). VEGF quantikine ELISA kits and goat anti-human VEGF_165_ antibody (AF-293-NA) were from R&D Systems. Phospho-VEGFR-2 (Tyr1175) Sandwich ELISA Kit was from Cell Signaling Technology. Mouse albumin ELISA kit (Bethyl Lab, Hamburg, Germany), paraformaldehyde (Merck, Darmstadt, Germany), histoclear (Biozym, Hessisch Oldendorf, Germany). VectaShield mounting medium (Vector Laboratories Inc., Burlingame, CA). Periodic acid (0.5%) and Schiff’s reagent (Merck), hematoxylin was from Fluka.

### Animal model

Male 10 weeks old 129/SV mice (Charles River, Germany) were held in individually ventilated cages and received a standard diet with free access to tap water. Weight-matched 129/SV mice received either 125 mg/kg body weight STZ (Sigma-Aldrich) in 50 mM sodium citrate (pH 4.5) or sodium citrate buffer (nondiabetic control group) intraperitoneally on day 1 and 4 for induction of hyperglycemia (glucose > 15 mmol/l). Mice received no insulin during the study. A total of 80 mice were used in this study. All procedures were performed according to the guidelines from the Federation of European Laboratory Animal Science Associations and were approved by local authorities (Lower Saxony State Departments for Food Safety and Animal Welfare); approval number 33.19-42502-04-15/1925.

Diabetic mice (n = 20) were treated for 12 weeks with placebo (saline), 100 mg/kg body weight CaD, or 30 mg/kg body weight enalapril by gavage. Body weight and glucose levels were measured every week. HbA1c (Olympus AU400) and Kidney function (serum creatinine) were measured at 6 and 12 weeks. Albuminuria was assessed using a mouse albumin ELISA kit (Bethyl Lab, Hamburg, Germany).

Sensory nerve conduction velocity (NCV) studies were performed in mice anesthetized with 2% isoflurane at week 6 and 12. Tail sensory NCV was determined by stimulating proximally along the tail at a recorded distance of 3 cm. For the measurement, a neuro-screen from Toennies Inc. was used. After 12 weeks of follow up the mice were euthanized by expose to isoflurane at 5% concentration which was continued for 1 min after breathing stop. Thereafter bilateral thoracotomy and laparotomy were performed and kidneys were perfused with ice cold saline solution via the left heart ventricle.

### Immunohistochemistry

For immunofluorescence, paraformaldehyde-fixed and paraffin-embedded tissue sections (2 μm) were processed as previously described [29]. After blocking with 10% rabbit serum, paraffin sections were stained with antibodies against pP38 and F4/80 and with a secondary antibody conjugated to Cy3. Specimens were analyzed using a Zeiss Axioplan-2 imaging microscope with AxioVision 4.8 software (Zeiss, Jena, Germany).

### Cell culture

Primary human umbilical vein endothelial cells (HUVECs) were routinely cultured in 0.1% gelatin pre-coated flasks or dishes, up to passage 6. The effect of CaD alone (0, 10, 20, 50, 100, and 200 μM) on cellular viability was assessed by CCK-8 kit using a Tecan Microplate Reader (Genios). CaD concentrations at 10 and 20 μM were not effective for the subsequent experiments so they were omitted. To measure the effect of CaD on VEGF-induced cell viability, HUVECs (1 × 10^4^ cells/well) were treated with VEGF (25 ng/ml) pre-mixed with various concentrations of CaD (0, 50, 100 and 200 μM) in medium without serum and growth factors (starvation medium) for 24 h and 48 h. The number of viable cells is presented relative to untreated controls. We did not observe a significant difference between cells treated with 100 or 200 μM CaD, therefore 200μM was not included in the subsequent experiments.

### Wound healing

Confluent HUVECs monolayer was scraped using a 0.2 ml pipette tip after 2 h of culture in starvation medium. Subsequently, cells were washed; fresh EGM medium containing 0.5% FCS and different concentrations of CaD (0, 50 and 100 μM) with or without 25 ng/ml VEGF was added. Images were taken using a Leica DM 14000B microscope after 16 h incubation. The gap distance of migrated cells was quantitatively evaluated using ImageJ software.

### Endothelial cell transwell invasion assay

The motility of HUVECs was performed in 24-well transwell plates using 8 μm polycarbonate filters coated with 0.1% gelatin. Cells were seeded into the upper chambers at a density of 1 × 10^5^ cells per chamber, the bottom chambers were filled with 600 μL 0.5% FCS EGM supplemented with VEGF (25 ng/ml) with or without CaD (0, 50, 100 μM). After 24 h, the number of migrated cells was evaluated with calcein-AM using Greiner bio-one quantitative cell migration assay protocol. The results were the means from 3 replicates of each experiment.

### Endothelial permeability assay

Permeability across endothelial cell monolayers was measured using gelatin-coated Transwell ThinCert^™^ 0.4 μm pore size polycarbonate filter in 24 well as previously described [30]. Briefly, HUVECs were plated at a density of 1×10^5^ cells per insert and were cultured for 48 hours until the formation of a tight monolayer. The cells were washed and then treated with VEG/CaD mixture for 2 h. After subsequent two washing steps, FITC-labelled dextran (1 mg/ml, molecular mass: 70 kDa; Sigma-Aldrich) was added to the upper compartments. After 2 h, 0.1 ml was collected from the lower compartment and fluorescence was measured using a Tecan Microplate Reader with 485/535 nm.

### Western blotting

Cells were seeded into 6 cm dishes till 80–100% confluency and then cultured in starvation medium for 2 h. For cells stimulated in the presence of heparin, CaD, VEGF and heparin were premixed for 1h at 37°C before addition to the cells. For heparanase (HPSE) treatment experiments, cells were starved for 1.5 h then treated for 30 min with HPSE at 37°C, washed 3 times with warm medium before addition of VEGF/CaD mixtures. For the interaction between CaD and VEGFR-2, the cells were first pre-incubated with CaD for 1 h in starvation medium, followed by washing with warm medium. Cells were then exposed to starvation medium supplemented with 25 ng/ml VEGF with or without the indicated CaD concentrations for 2 min (pVEGFR2), 15 min (downstream signaling molecules) or 2 h (tight junction proteins). The optimal time points were determined by our preliminary experiments. Cells and mouse kidney tissue were lysed in RIPA buffer containing 1 mM PMSF, 1 mg/ml aprotinin, 1 mg/ml leupeptin, 1 mM Na3VO4, 1 mM NaF and incubated for 15 min at 4 °C. Cell lysates and tissues were centrifuged at 10 000 rpm for 10 min and 30 min respectively. Protein concentrations were determined using a Bradford protein assay kit (Bio-Rad, Germany). Proteins (50–100 μg) were applied to 7.5%-15% SDS PAGE gels and transferred onto a PVDF membrane (Millipore, USA). The membranes were incubated with specific primary antibodies. Protein bands were detected using an enhanced chemiluminescence method. Bands were normalized with GAPDH or vinculin as an internal control. Expression of phosphorylated proteins was normalized to the level of total protein expression.

### Immunofluorescence and enzyme-linked immunosorbent assay (ELISA)

HUVECs were seeded at 1 × 10^4^ cells/well onto coverslips in a 12-well plate until 60% confluent. Serum-starved cells were treated with or without 100 μM CaD/25 ng/mL VEGF for 15 min (for actin filaments detection) or 2 hours (for ZO-1 expression). The cells were then fixed in 4% paraformaldehyde and then permeabilized with 0.5% Triton X-100 (for ZO-1 staining the cells were not permeabilized). Actin filaments were stained by phalloidin-Alexa fluor 488 (1:250) for 1 h at room temperature and nuclei were detected by DAPI. Images were captured by Leica DM 1400B confocal microscope. For ZO-1 detection, the cells were stained with ZO-1 antibody (1:100) and subsequently with Alexa fluor 488 secondary antibody (1:250). The slides were examined with Olympus Fluoview FV1000 confocal laser scanning microscope system (Olympus, Tokyo, Japan). The concentration of VEGF and pVEGFR-2-Tyr1175 in mouse kidney lysates was measured using commercially available ELISA kits.

### Quantitative RT- PCR analysis

mRNA from kidney sections in RNA later was isolated using RNeasy miniprep kit (Qiagen). qPCR was performed on a LightCycler 96 Real-Time PCR System using SYBR Green RT-PCR with the following Quantitec primers from Qiagen; IL-6 (QT0009887), CXCL1 (QT00113253), MCP-1 (QT00167832), IL-1ß (QT01048355) and TNF-α (QT00104006). Quantification was carried out by LightCycler 96 software and the amount of RNA was expressed as fold change relative to the housekeeping gene (β-Actin; QT00095242).

### Solid-phase binding assay of biotinylated VEGF to recombinant human VEGFR1–2

The method was performed as described previously [31]. Briefly, 96-well microplate was coated with 500 ng/ml of either VEGFR-1 or -2 in PBS, sealed and incubated overnight at 4°C. After 3 times washes with PBS-Tween 20 (0.05% v/v), the plate was blocked with PBS with 1% (w/v) BSA, and incubated for 2 h at room temperature. After washing, a mixture of bt-VEGF (50ng/ml) and heparin (1 μg/ml) or various concentrations of CaD in PBS were applied overnight at 4°C. After washing, streptavidin-HRP (1:4000) was added for 2 h, washed and substrate solution was added for 30–45 minutes. Stop solution (2N; H_2_SO4) was added and fluorescence was measured at 450nm.

### Proximity ligation assay

HUVECs were treated with VEGF/CaD for 2 min (time point was chosen based on the optimal VEGFR-2 phosphorylation signal observed in immunoblotting) followed by fixation in paraformaldehyde and permeabilization as described above. Blocking and Proximity ligation was performed using a Duolink PLA kit according to the manufacturer’s protocol. To study VEGF/VEGFR2, VEGF/HS and VEGFR2/HS interactions, cells were incubated overnight at 4 °C with goat monoclonal VEGF (1:100) and/or mouse monoclonal heparan sulfate (1:200), rabbit polyclonal anti-VEGFR2 (1:800). All images were taken with a Leica DMI3000 B microscopy with a 20x objective and analyzed with NIH ImageJ software.

### Statistics

All data are expressed as the mean ± SD of indicated *n* values (for *in vitro* data) and mean ± SEM (for *in vivo* data). One-way analysis of variance was used to compare between groups. Data were analyzed using post hoc Bonferroni correction for multiple comparisons. P-values are *(P<0.05), ** (P<0.01), *** (P<0.001).

## Results

### CaD inhibits VEGF-induced VEGFR-2 phosphorylation in HUVECs

**Fig 1:**
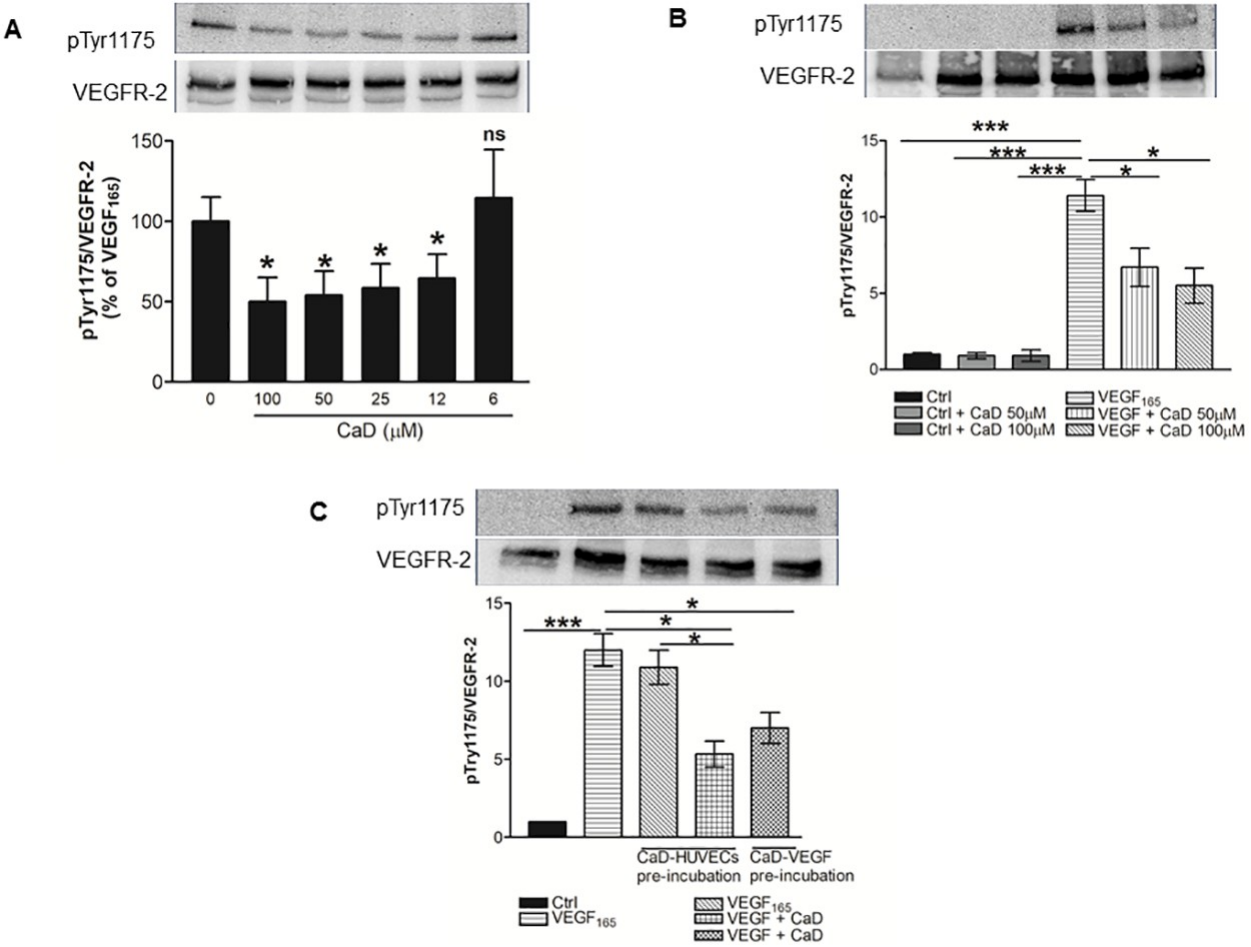
Calcium dobesilate (CaD) inhibits VEGF-induced VEGFR-2 activation. VEGF_165_ (25 ng/mL) was premixed and incubated with: (**A**) various CaD concentrations (6–100 μM) or (**B**) 50–100 μM CaD for 1 h, before exposure to HUVECs for 2 min. (**C**) HUVECs were incubated with 50 and 100μM CaD for 60 min with three subsequent washing steps with warm medium before stimulation with VEGF_165_ for 2 min. Western blot analysis was performed using anti-phospho-VEGFR-2 antibody and total VEGFR2 was used as a loading control after membrane stripping. Each bar represents the mean ± SD (n = 3). *P<0.05, ***P<0.001 versus VEGF-treated HUVECs.

The cells were treated with a range of CaD (0–200 μM) for 24 h and 48 h and assayed by Cell Counting Kit-8. The cells exhibited no signs of cytotoxicity (S1 Fig). We then evaluated the inhibitory effect of CaD on VEGF-induced activation of the VEGFR-2 signaling pathway and the angiogenic response in HUVECs. Firstly, serial dilution of CaD (6–100 μM) was conducted to determine the optimal concentration that could inhibit VEGFR-2 phosphorylation. CaD was pre-mixed with VEGF_165_ (25 ng/mL) for 60 min prior to being added to HUVECs. CaD significantly decreased VEGF-induced VEGFR-2 phosphorylation in a concentration-dependent manner (Fig 1A). CaD reduced VEGF-induced phosphorylation of VEGFR-2 up to 50% without affecting the overall VEGFR-2 expression level (Fig 1B, lanes 5 and 6). These results suggest that, either CaD binds directly to VEGF molecules and inhibits its binding to the VEGFR-2 or CaD in the medium is competitively inhibiting recruiting of VEGF to cell surface.

Secondly, HUVECs were pre-incubated with CaD (100 μM) for 1 h. Subsequently, the cells were rinsed, VEGF_165_ (25 ng/mL) was added for 2 min. Pre-incubation of cells with CaD did not significantly reduce VEGFR-2 activation after VEGF stimulation (Fig 1C, lane 3) suggesting that the inhibitory effect of CaD on VEGFR-2 phosphorylation is not mediated by the direct binding of CaD to cell surface components of endothelial cells.

In the third setup, both cells and VEGF_165_ were pre-incubated with CaD to investigate for possible dual effect of CaD on the ligand and the receptors. We observed an additive effect of CaD inhibition of VEGFR-2 signaling (Fig 1C, lane 4). The inhibitory effect of CaD seems to be mainly mediated by the direct binding of CaD to VEGF or by competitive inhibition and to a lesser extent interacting with the cell surface components of the endothelial membrane.

### CaD attenuates VEGF-induced phosphorylation of MEK/ERK1/2 MAP kinase

**Fig 2:**
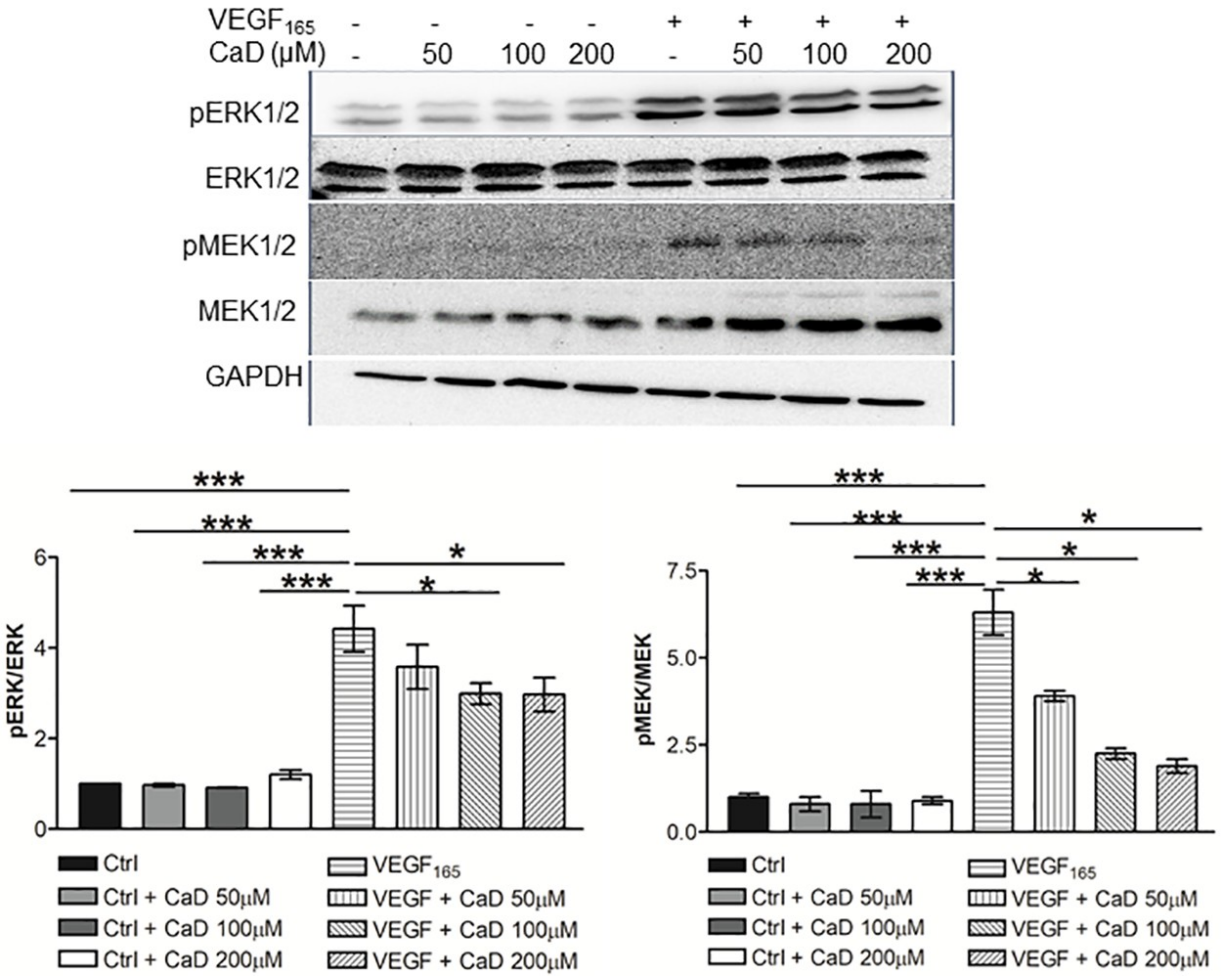
Calcium dobesilate (CaD) inhibits VEGF-induced MEK/ERK MAP kinase activation. CaD at 50, 100 and 200μM was incubated with VEGF (25 ng/mL) for 60 min before exposure to HUVECs for 15 min. Phosphorylated ERK and MEK1/2 was determined by Western blot as described in Figure 2. Data are expressed as mean ± SD (n = 3). * p < 0.05, *** p < 0.001, significantly different from VEGF-treated HUVECs.

We furthermore investigated the effect of CaD on the VEGF-induced signaling cascade [32]. Treatment with VEGF_165_ induced a strong phosphorylation of the ERK1/2 MAP kinase (Fig 2 lane 5). Co-treatment with CaD (100 and 200 μM) attenuated VEGF_165_-induced phosphorylation of ERK1/2 by 40% (Fig 2, lanes 7–8). The MAPK kinase MEK1/2 is known to be the direct upstream kinase of the ERK1/2 MAP kinase. We next investigated the effects of CaD on this signaling molecule upstream of the ERK1/2 MAP kinase, CaD markedly attenuated VEGF_165_ stimulated phosphorylation of MEK1/2 (Fig 2).

### CaD inhibits VEGF-induced angiogenic activity in HUVECs

**Fig 3:**
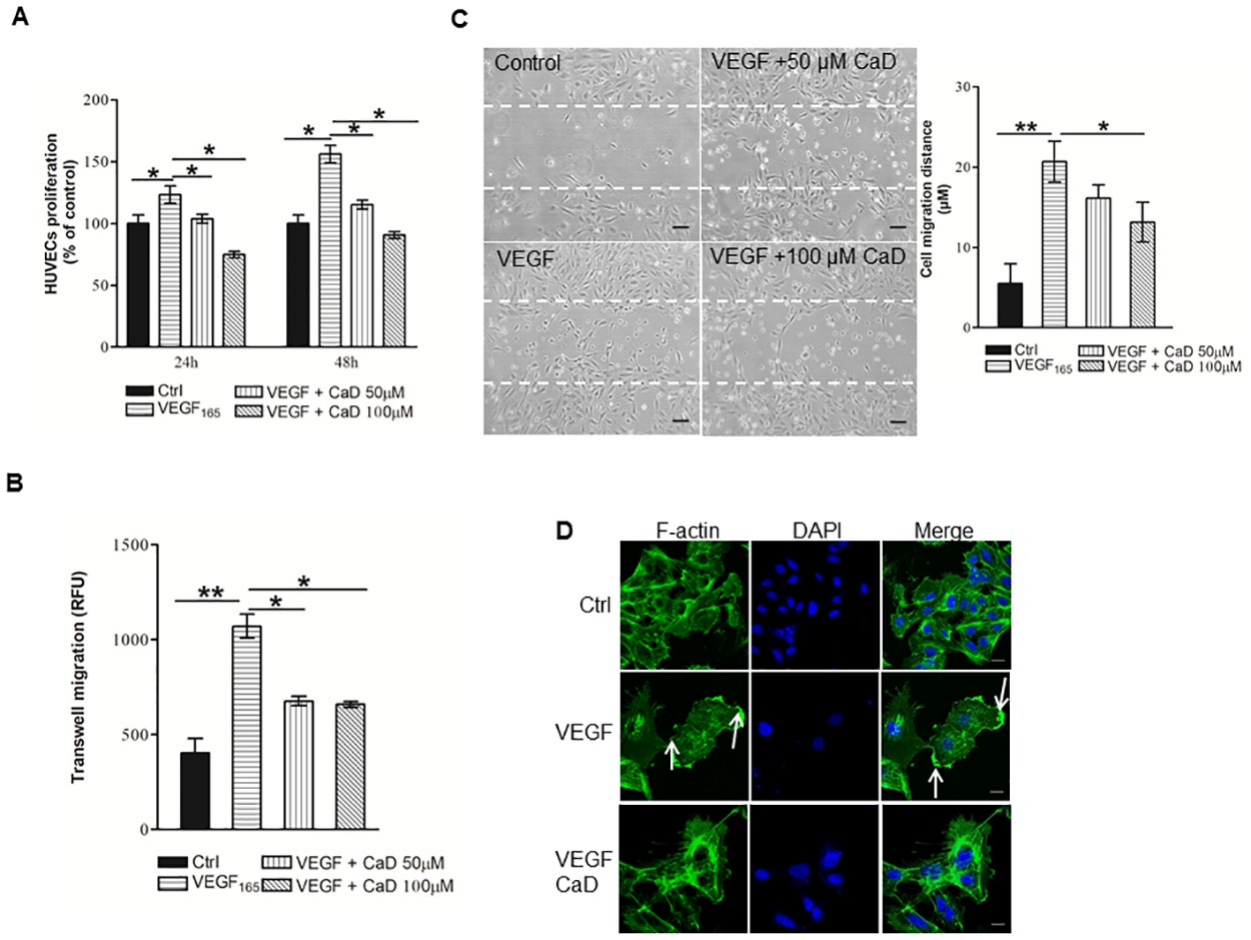
Inhibition of endothelial cell proliferation, invasion and migration by CaD. HUVECs were plated in 96-well plates, allowed to attach overnight, and then cultured for 24 h-48 h with CaD in the presence of VEGF_165_ (25 ng/ml)-CaD mixture (A) at the indicated concentration. The proliferation was measured as described in the Materials and Methods section. (B) Serum-starved HUVECs were allowed to migrate through trans-well membranes towards a vehicle, VEGF_165_ (25 ng/ml) and/or CaD at the indicated concentration for 24 h. Cells that had migrated to the underside of the membrane were processed for calcein-AM staining as described in the materials and methods section. (C) A monolayer of HUVECs was scratched and fresh medium containing vehicle, VEGF and/or CaD was then added. After 14 h, migration distance of HUVECs was quantified. Original magnification, 40x. (D) HUVECs were incubated with vehicle, VEGF_165_ (25 ng/ml) and CaD for 15 min. Cells were stained with phalloidin-Alexa Fluor 488 (left images) and DAPI (middle images). Merged view is represented in the right images. Original magnification, 40x. Scale bars represent 5 μm. The results shown are the means ± SD of four independent experiments conducted in triplicate. *P<0.05, **P<0.01, versus VEGF_165_-treated HUVECs.

Treatment with CaD significantly inhibited VEGF-induced proliferation and migration of HUVECs (Fig 3A–3C). Peripheral accumulation of F-actin was detected only in VEGF-stimulated cells (Fig3D middle panels). Treatment with CaD completely abrogated VEGF induced accumulation of peripheral actin-rich lamellipodia-like structures (lower panels).

### Effect of CaD on VEGF_165_- induced tight junction disruption and permeability

**Fig 4:**
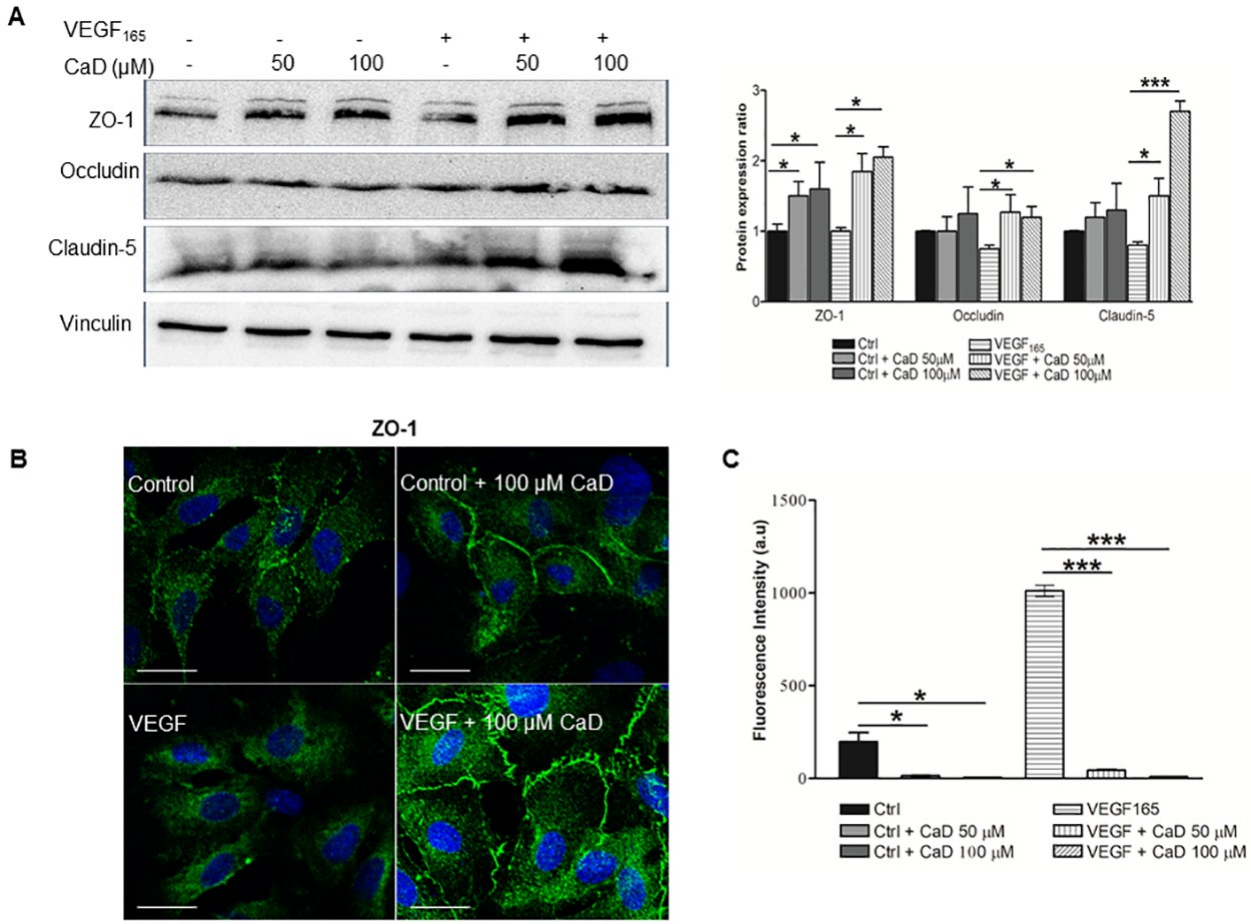
CaD prevents the decrease in tight junction protein levels and the increase in HUVECs permeability induced by VEGF_165_. CaD at 50 and 100 μM was incubated with VEGF (25 ng/mL) for 60 min before exposure to HUVECs for 2 h. Tight junction protein levels were determined by Western blotting (A), immunofluorescence (B) and endothelial cells permeability was by FITC-Dextran assay (C) as described in material and methods section. Representative images are shown and each bar indicates the relative expression to that of control or VEGF_165_ alone. Scale bars represent 25 μm and the average of three independent experiments ± SD. *P<0.05, ***P<0.001.

The tight junction proteins occludin, claudin-5 and ZO-1 were expressed by HUVECs as shown by Western blot analysis. Treatment with CaD significantly prevented the decrease in ZO-1, Occludin and claudin-5 expression induced by VEGF (Fig 4A, lanes 5 and 6). The expression of ZO-1 and claudin-5 proteins was dependent on the concentration of CaD. ZO-1 is known as a central regulator of intercellular junctions in endothelial cells, controls endothelial adherence junctions, barrier formation, angiogenesis as well as regulation of endothelial permeability [33,34]. Therefore, we further investigated ZO-1 expression by immunofluorescence. Labelling of confluent control cells showed a moderate junctional staining at cell boarders Fig 4B (upper left panel; arrows), whereas VEGF-treated cells lacked junctional staining and showed diffused ZO-1 staining (lower left panel; arrowheads). Moreover, when ZO-1 labelling of control cells and those treated with VEGF was compared with that of CaD-treated cells it was evident that, CaD-treated cells (right panels) not only maintained, but also increased their junctional assembly of ZO-1 compared with controls and VEGF treated cells. To test for the effect of CaD on endothelial permeability, VEGF/CaD was added to the basolateral sides of HUVECs confluent monolayers, cells were cultured for an additional 2 hours, and then processed for dextran permeability. VEGF treatment caused a significant increase in FITC-dextran flux which was significantly decreased by CaD treatment (Fig 4C).

### Mechanism of action of CaD inhibitory effect on VEGF_165_

**Fig 5:**
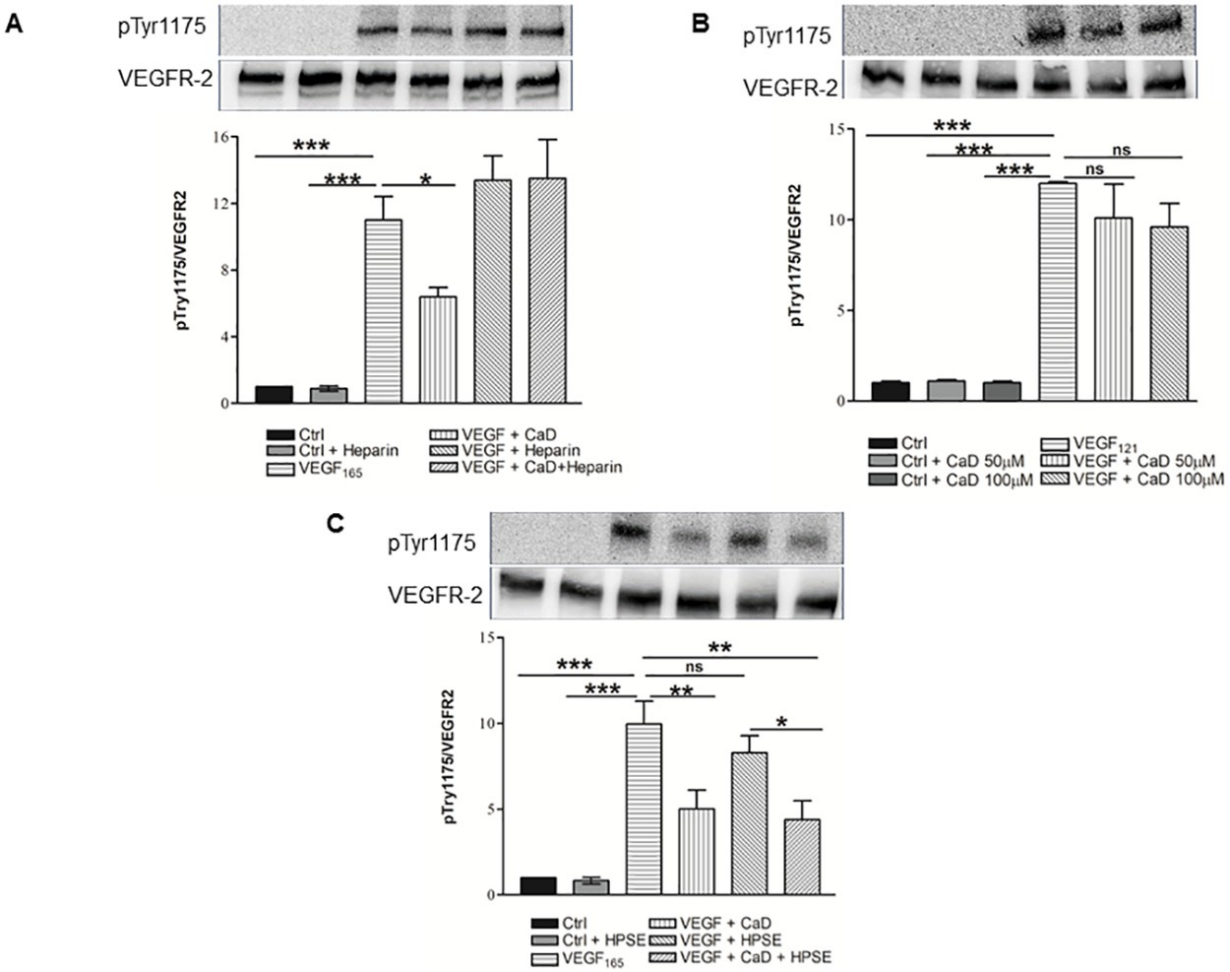
Effect of heparin and heparanase (HPSE) treatment on the inhibitory effect of CaD. (A) VEGF_165_ (25 ng/mL), CaD (100 μM) and heparin (10 μg/ml) were pre-mixed and incubated at 37°C for 60 min prior to HUVECs stimulation for 2 min. (B) HUVECs were stimulated with VEGF_121_ (25 ng/ml)-CaD mixture as described in Fig 1 for 2 min. (C) HUVECs were digested with HPSE (0.5 μg/ml). After 30 min, each dish was washed and then stimulated with 25 ng/ml VEGF_165_ alone or VEGF-CaD (100 μM) mixture for 2 min. Samples were subjected to gel electrophoresis, and Western blotting was performed as described. Each bar indicates the relative phosphorylation to that of VEGF alone or VEGF-HPSE and the average of at least three independent experiments ± SD. *P<0.05, **P<0.01, ***P<0.001.

CaD (100 μM), VEGF (25 ng/ml) and heparin (10 μg/ml) were premixed for 1 h before addition to the HUVECs for 2 min. Phosphorylation of VEGFR-2 was examined by Western blot analysis. As revealed by Western blot analysis, the VEGF_165_-induced phosphorylation of VEGFR-2 increased in the presence of heparin (Fig 5A, lane 5). Heparin abrogated CaD inhibitory effect on VEGFR-2 phosphorylation (Fig 5A, lane 6). For comparison, we examined the effects of CaD on VEGF_121_, an isoform without heparin binding domain (HBD) [35]. CaD did not significantly inhibit VEGF_121_-induced tyrosine phosphorylation of VEGFR-2 (10%) (Fig 5B, lanes 5 and 6).

### CaD inhibits VEGF_165_-induced phosphorylation of VEGFR-2 in heparanase (HPSE) treated cells

Previous studies showed that the capacity of VEGF_165_ to bind its receptors on endothelial cells was abolished by HPSE treatment and that the effect of HPSE could be reversed by the addition of heparin [36,37]. Cells were treated with or without HPSE (30 min), then washed and stimulated with CaD-VEGF_165_ for 2 min. VEGF_165_-induced phosphorylation of VEGFR-2 was reduced to 75% by HPSE treatment (Fig 5C, lane 5) suggesting that VEGF-induced receptor phosphorylation is dependent in part on the presence of the heparan sulfates [36,38]. Interestingly, the decrease in VEGF_165_-induced phosphorylation by digestion with HPSE was further decreased to 50% by CaD (Fig 5C, lane 6).

### CaD inhibits formation of VEGF_165_ -VEGFR-1/2 or VEGF-heparan sulfate complexes

**Fig 6:**
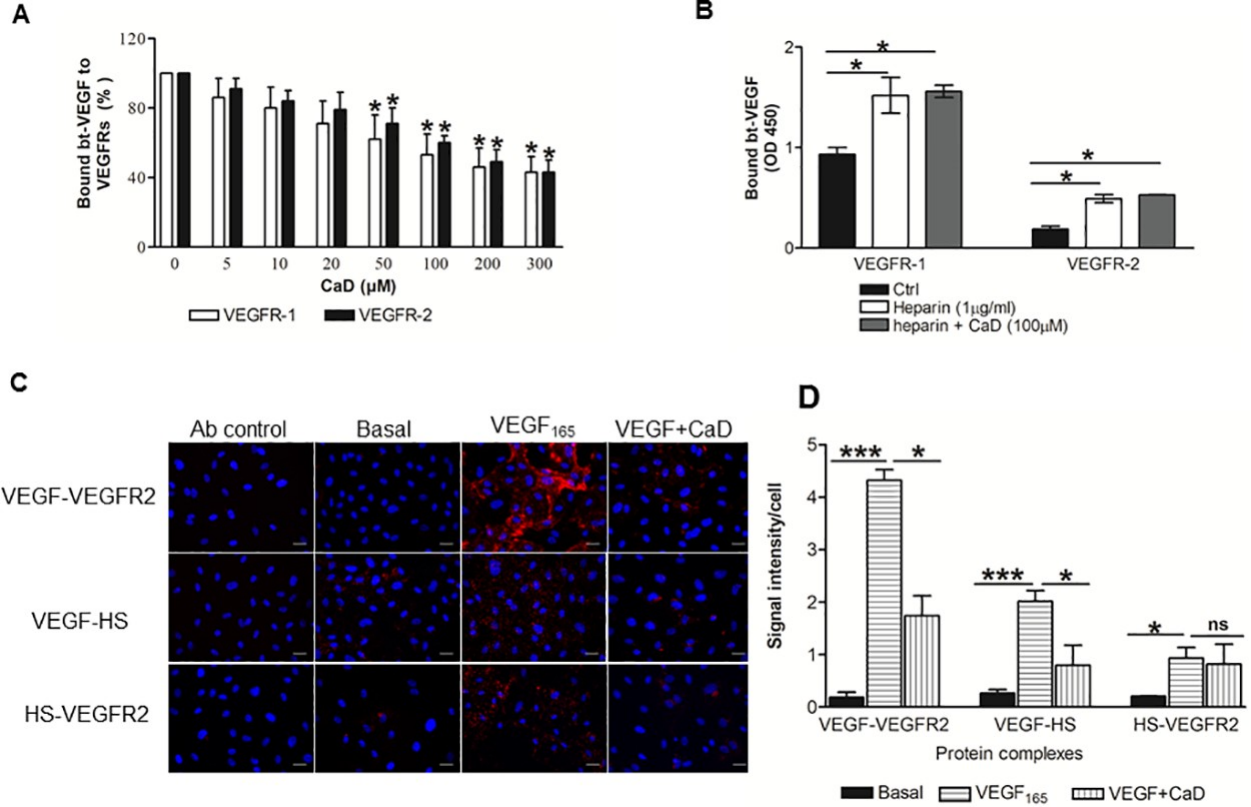
Effect of CaD on VEGF_165_ binding to VEGFR-1/2, heparin and HS. (A) bt-VEGF_165_ binding assay to immobilized VEGR-1/2 in the presence of increasing concentrations of CaD. Data are expressed as percentage of control (no CaD) ± SD in binding from four independent experiments; *p < 0.05 vs. no CaD. (B) bt-VEGF_165_ binding in the absence or presence of 1 μg/ml heparin or CaD (100 μM) to VEGFR-1 and VEGFR-2. Data are expressed as mean OD at 450 nm ± SD from four independent experiments; *p < 0.05 vs. no heparin or CaD (Ctrl). (C) Representative images were obtained by proximity ligation assay. Cells were fixed and incubated with antibodies to human VEGF (goat IgG) and to human VEGFR-2 (rabbit IgG) (upper panel) or to VEGF and heparin sulphate (mAb 10E4) (middle panel) or to VEGFR-2 and HS (lower panel) followed by proximity ligation assay reagents. Each red dot indicates a protein interaction. Nuclei are shown in blue. The antibody (Ab) control panel represents cells incubated with the corresponding IgG. (D) Quantified data presented as red dots (signal intensity)/cell. The error bar represents SD. n = 3 separate experiments *P<0.05, ***P<0.001 relative to that of VEGF alone. All images were taken with a Leica DMI3000 B microscopy, scale bar 100 μm and analyzed with NIH ImageJ software.

Because both VEGF-A_165_ and VEGFR1/2 bind heparin [39,40], exogenous heparin may also play a cross-bridging role in the engagement of the protein ligand with its receptor and CaD could destabilize this complex. We performed an *in vitro* cell-free solid phase binding assay for both VEGFR-1 and VEGFR-2. CaD concentration-dependently inhibited biotinylated-VEGF_165_ (bt-VEGF_165_) binding to VEGFR-1/2 (Fig 6A). In conformity to the previous studies, heparin increased the binding of bt-VEGF_165_ to VEGFR-1/2 at lower concentrations (0.01–1 μg/ml) [38,41,42], whereas higher heparin concentrations (10–1000 μg/ml) inhibited bt-VEGF binding to the receptors (S2 Fig). In the presence of heparin (1 μg/ml), CaD inhibitory effect on bt-VEGF_165_ binding to VEGFRs is abrogated (Fig 6B). As expected Duolink *in situ* proximity ligation assay (PLA) further confirmed the inhibited interaction between VEGF-VEGFR-2 and between VEGF-HS in the presence of CaD (Fig 6C). Quantification of the PLA signal revealed a 60% decrease in VEGF-VEGFR-2 and VEGF-HS complexes in the presence of CaD but only 12% decrease in HS-VEGFR-2 complexes (Fig 6D). Our data further suggest that CaD interferes with HS binding to the ligand and not to the receptors.

### Protective effects of CaD treatment in a type I diabetes mouse model

**Fig 7:**
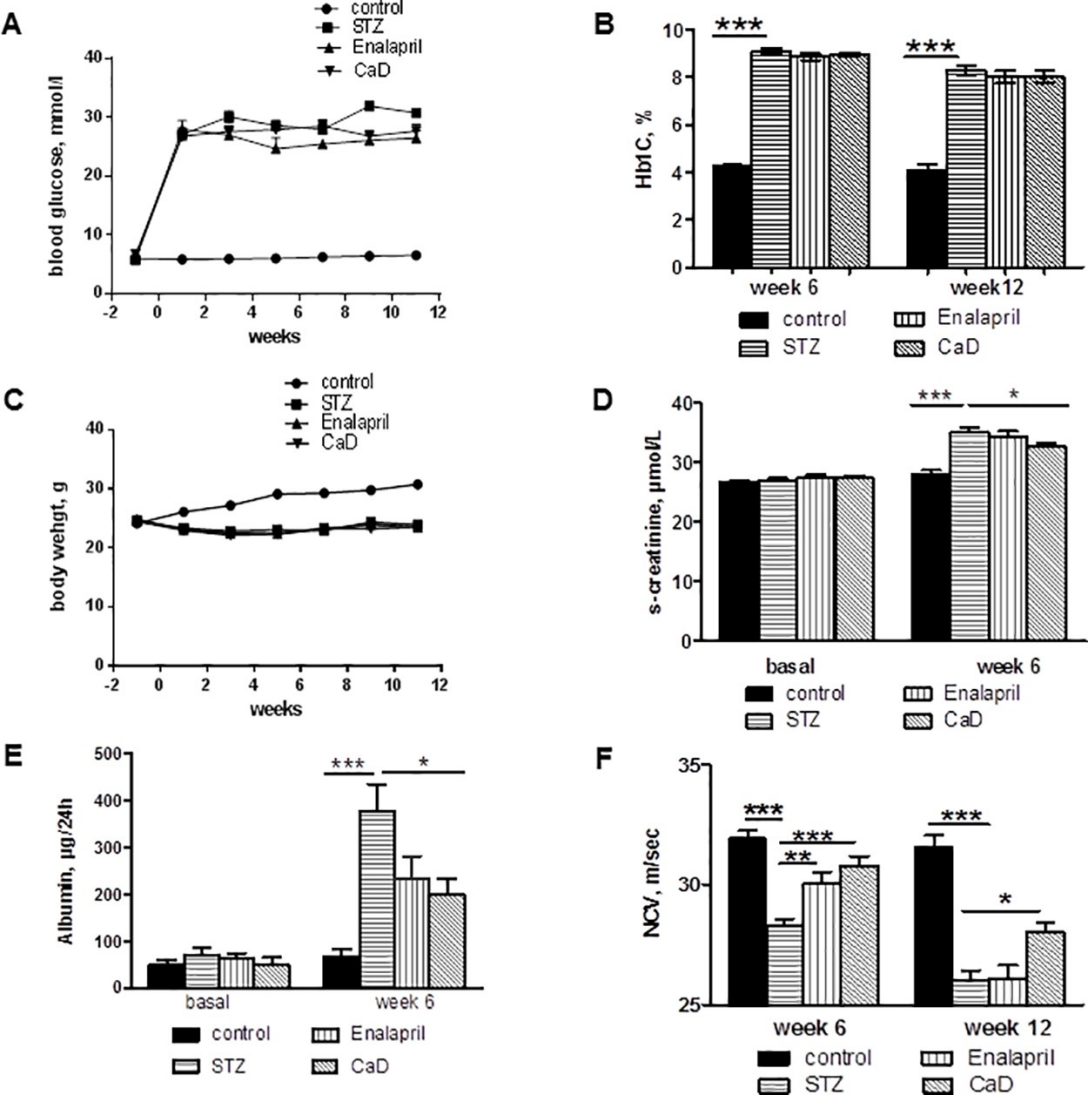
Effect of CaD treatment on diabetic complications in vivo. Type I diabetes was induced in Sv129 mice and thereafter the diabetic mice were daily treated with vehicle or CaD (100mg/kg) or enalapril (30mg/kg). CaD treatment had no effect on hyperglycemia (A and B) and body weight (C). CaD treatment protects kidney function in diabetic mice as reflected by serum creatinine levels (D) and albuminuria (E). CaD treatment reduces diabetic neuropathy as reflected by sensory nerve conduction velocity measurements (F). The results are expressed as the mean+SEM (n = 20 mice for each group). *p<0.05, **p<0.01 and ***p<0.001 versus vehicle treated diabetic mice (STZ).

The effect of CaD compared to enalapril treatment was further investigated in vivo using type I diabetes mouse model (STZ-induced diabetes). Treatment with CaD/enalapril had no effect on glucose levels and body weight in diabetic mice (Fig 7A–7C). CaD but not enalapril significantly reduced diabetic nephropathy as reflected by serum creatinine levels (Fig 7D) and albuminuria (Fig 7E). CaD treatment also reduced diabetic neuropathy. After 6 week diabetes a reduction of the sensory nerve conduction velocity was observed in STZ/vehicle and STZ/enalapril groups but not in STZ/CaD group. At week 12 decreased nerve conduction velocity was observed in all STZ groups compared to nondiabetic controls, but in the STZ/CaD group the decrease was significantly less pronounced (Fig 7F).

**Fig 8:**
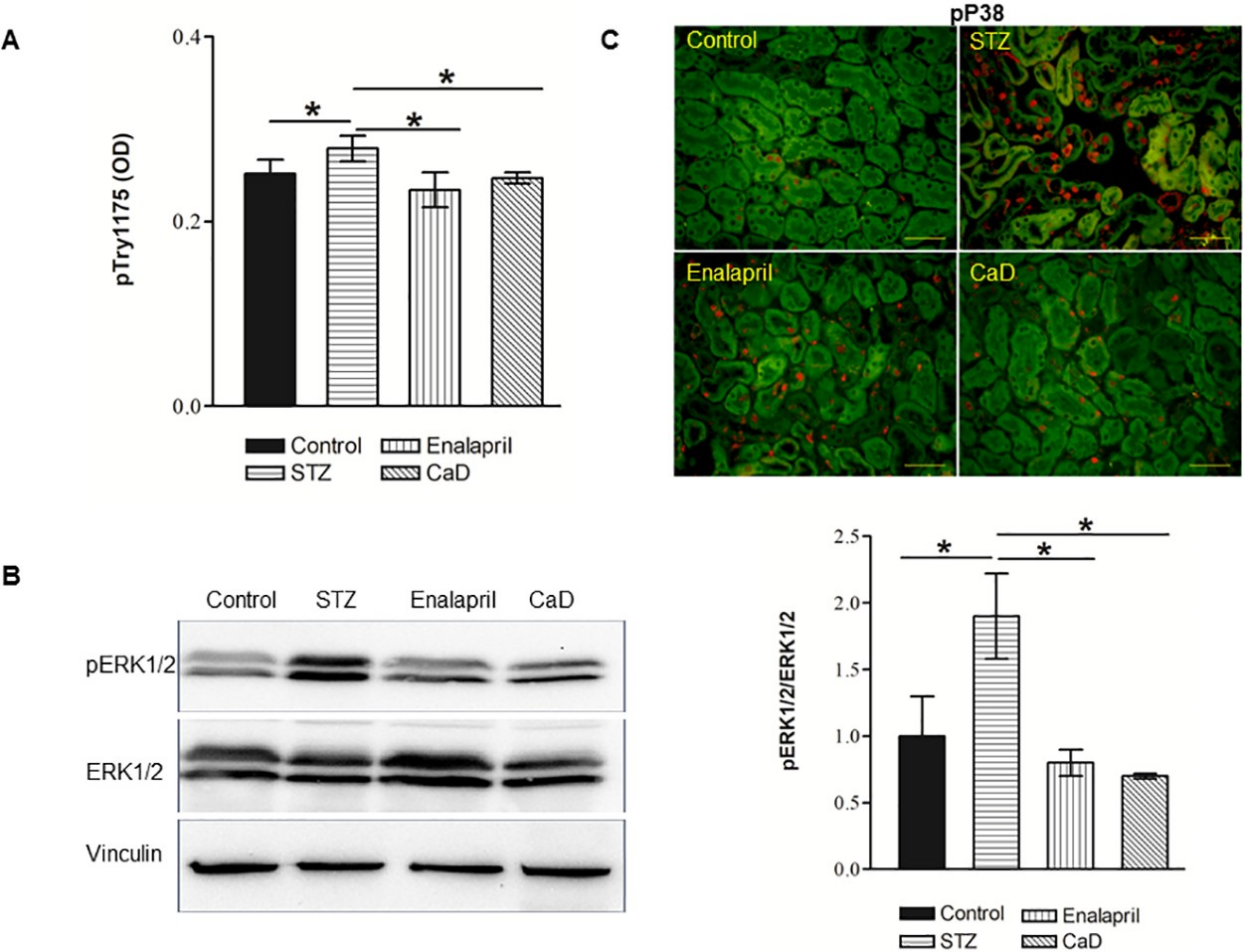
CaD inhibits VEGFR-2 phosphorylation and signaling in STZ diabetic mice. STZ mice were daily treated with vehicle, CaD or Enalapril and sacrificed at week 12 for the analysis. Non diabetic mice were used as control. Kidneys were isolated and homogenized as described in materials and methods. Phosphorylated VEGFR-2 (A) in the kidney lysates was determined by ELISA mean ± SD of 5–6 animals. Phosphorylated ERK1/2 (B) was analyzed by Western blot as described in Fig 1. Vinculin was used as a loading control. Phosphorylated P38 (C) was analyzed by immunohistochemistry as described in the materials and methods section. Autofluorescence is shown in green, scale bar 100 μM. Representative image for n = 6/condition is depicted. *P<0.05 versus vehicle treated STZ mice.

**Fig 9:**
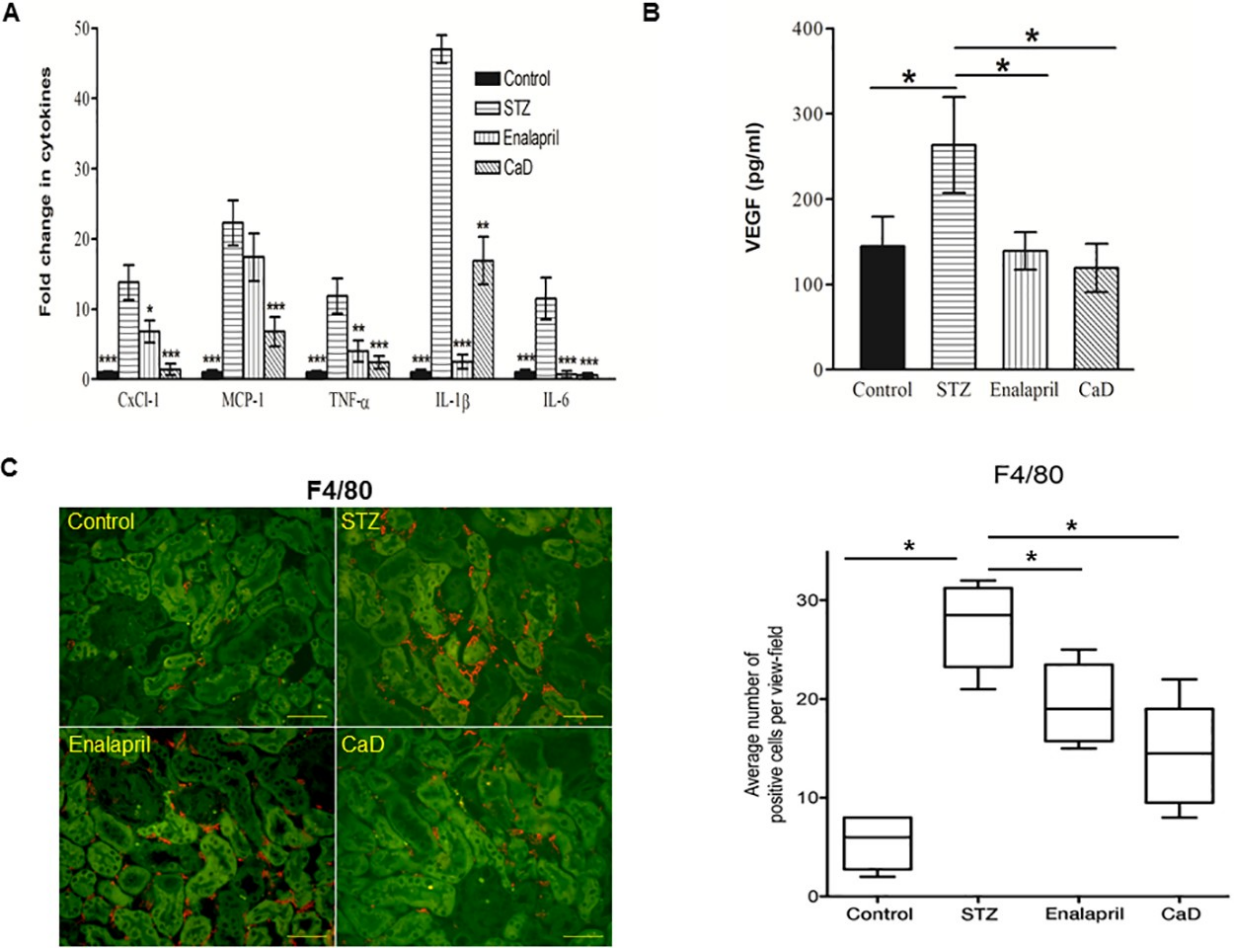
CaD prevents the increase in diabetes-induced renal inflammation. The mRNA levels were assessed by real time PCR and normalized to Actin (A). VEGF production was determined by VEGF_164-188_ ELISA according to the manufacturer’s instructions (B). Data represent the mean ± SD of 4–8 animals. * p < 0.05. Significantly different from vehicle treated STZ mice. Immunohistochemistry for F4/80 (red) in kidney cross sections of non-diabetic (control), diabetic vehicle treated (STZ), enalapril and CaD treated STZ mice (C). Autofluorescence is shown in green, scale bar 100 μm. Images are exemplary for n = 5/condition. Infiltrating cells were quantitatively analyzed by scoring five areas in each kidney section for F4/80-positive cells. Each bar represents the mean ± SD.* p < 0.05, **P<0.01, *** p < 0.001. Significantly different from vehicle treated STZ mice.

We next investigated the effects of CaD on diabetes-induced VEGF signaling in the kidney. CaD/enalapril treatment decreased p-VEGFR2 level in the kidney compared to the vehicle-treated diabetic mice (Fig 8A) and significantly suppressed diabetes-induced ERK1/2 (Fig 8B, lane 4 and column 4) and P38 phosphorylation (Fig 8C). It was accompanied by reduced inflammation in the diabetic kidneys as reflected by prevented up-regulation of CXCL-1, IL-1ß, TNF-α and IL-6 expression (Fig 9A). Whereas both CaD and enalapril were effective for these four cytokines, only CaD but not enalapril inhibited significantly MCP-1 up-regulation (Fig 9A). Moreover, diabetes-induced up-regulation of VEGF in the kidney was down-regulated by CaD/enalapril treatment (Fig 9B). In line with reduced levels of pro-inflammatory mediators, an increased amount of F4/80 positive macrophages in the interstitial areas of diabetic kidney was significantly reduced in CaD treated mice (Fig 9C).

## Discussion

CaD is used in particular in Asia and South America to treat diabetic retinopathy [23], chronic venous insufficiency, and various conditions associated with excessive angiogenesis [43,44]. Recent studies suggested that CaD exerts protective effects against diabetic nephropathy [45,46]. Despite its broad use, the pharmacology of CaD has received little attention. We showed that CaD significantly blocked VEGF and diabetes-induced VEGFR-2 phosphorylation, which is the main mediator of proliferation, migration, survival, and permeability in endothelial cells [47]. Earlier studies showed that the MAPK signaling cascade, ERK and P38, were also modulated via VEGFR-2 signaling activation by VEGF on HUVECs [48–50]. We therefore, investigated the effect of CaD on VEGF and diabetic-induced ERK1/2 and P38 phosphorylation in HUVECs and in our mouse model respectively. We also monitored the effects on VEGF-induced endothelial cell proliferation, invasion, and migration and found these components relevant for novel blood vessel formation to be significantly inhibited in the presence of CaD. Furthermore, we found that CaD significantly down-regulated VEGF_165_- induced phosphorylation of ERK1/2 and diabetic-induced phosphorylation of ERK1/2 and P38. Similar results have been reported, in which CaD significantly inhibited FGF-induced ERK phosphorylation in glioma cells and P38 in diabetic retinopathy [51–53].

Consistent with the anti-migratory function, CaD also abolished VEGF-induced polymerization of actin in lamellipodia-like structures. Zhou and colleagues have recently demonstrated an inhibition of endothelial cell proliferation and migration by CaD under hyperglycemic conditions [25], which they attributed to the corresponding changes in VEGF expression. Instead, we demonstrate a direct effect of CaD on VEGF signaling. Our findings complement those of Angulo et al., who demonstrated a significant reduction in VEGF-induced HUVECs proliferation by CaD [32]. These findings support an important role for dobesilate in vascular angiogenesis.

Dysfunction of the endothelial tight junction is crucial for the development of endothelial hyper-permeability [54]. We tested the effect of VEGF and CaD on endothelial cells tight junction proteins and permeability. CaD significantly restored VEGF-induced suppression of ZO-1, Occludin and Claudin-5 expression and VEGF-induced increased permeability, indicating that the protective effects could be related to stabilizing tight junction proteins and therefore, suggesting a potential of CaD to improve endothelial integrity. Moreover, both immunoconfocal and FITC-Dextran permeability analysis revealed that CaD-treated cells exhibit not only strong and continuous ZO-1 presence at the membrane, but also a corresponding significant decrease in permeability. This was confirmed by immunoblotting, which demonstrated increased ZO-1 expression. These observations suggest that the addition of CaD obviously restored cell structure and stabilized morphological characteristics of ZO-1 and increased its synthesis in endothelial cells, but further studies will be needed to fully elucidate the role of CaD-VEGF interaction in the expression of ZO-1 in endothelial cells.

We propose that the dominant mechanism of CaD resulting in the inhibition of VEGF-induced VEGFR-2 activation and signaling is related to the interaction between CaD and HS. Earlier studies demonstrated that CaD interferes with heparin binding on FGF and inhibits the signaling of FGF via its receptors FGFRs [27,28]. We treated HUVECs with a mixture of VEGF_165_, CaD, and heparin and found that the inhibitory effect of CaD on VEGFR-2 phosphorylation was abrogated by the addition of heparin. By using an *in vitro* cell-free ELISA and proximity ligation assays, we show that CaD clearly interfered with the binding of VEGF to its receptors and also to HS the effect of CaD was accordingly overridden by the addition of heparin. These findings suggest that, binding of CaD to VEGF probably lowers the affinity of VEGF to its cognate receptors because of change in three-dimensional structure of VEGF at its receptor recognition site, and/or dissociating the receptor-growth factor signaling complex as previously suggested for FGF [27]. In contrast to our cell-free ELISA assay where we show that heparin enhanced bt-VEGF165 binding to VEGFR-1, previous studies reported a minimal or non-effect of heparin on VEGFR-1 binding ([41,55]. This could be explained by the higher heparin concentration (10 μg/ml) used in their ELISA assay. In this study we observed an inhibition of VEGF binding to VEGFR-1 and -2 in cell-free based ELISA assay in the presence of high heparin concentrations (more than 1μg/ml). Similar observations have been reported in cell-free and cell-based assays with respect to VEGFR-2 [38,39,42]. Therefore it is most likely that the same phenomenon applies for VEGFR-1 although further studies are required to substantiate this hypothesis. To further substantiate the involvement of heparin binding domain in the interaction with CaD, HUVECs were stimulated with VEGF_121_, an isoform without the exon-7-encoded region, which has no capacity to bind to heparin. As expected, CaD did not significantly inhibit VEGF_121_ induced receptor phosphorylation. We suggest that CaD forms a complex with VEGF_165_ and VEGFR-2, thereby inhibiting VEGF_165_-dependent signaling. HS/heparin has been proposed to regulate VEGF biological activity by binding VEGF directly [56], and also by interacting with receptors [39,40]. A report by Fernandez and colleagues demonstrated the dual inhibitory action of CaD in endothelial cells by binding to both FGF and its receptors [27]. Our results suggest that the possible mechanism of the CaD action is related to interaction with heparin binding VEGF_165_ and to a lesser extent to the VEGF receptors. This interpretation is further supported by our findings using non-heparin binding VEGF_121_, where CaD did not significantly affect VEGF_121_-induced VEGFR-2 phosphorylation and also by our PLA where CaD did not prevent HS-VEGFR-2 complex formation.

To further substantiate our findings, the role of cell surface HS in VEGF_165_ activity was assessed by the reduction of VEGF_165_-induced phosphorylation of VEGFR-2 by 25% in the cells digested with HPSE, this minimal reduction was also observed by Ashikari et al., even though the percentage of HS removed from the cell surface was reduced to less than 5% [36]. Accordingly, our recent study demonstrates a significant reduction in surface endothelial cells HS after HPSE digestion [57]. Interestingly, CaD treatment further reduced VEGF-induced phosphorylation of the receptor in HPSE treated cells to 50%, similar to the level observed in CaD treatment without HPSE suggesting that in addition to CaD inhibiting VEGF binding to surface HS, there is a percentage of inhibition which is contributed by CaD inhibiting VEGF binding to VEGFR-2 that has to be taken into account.

**Fig 10:**
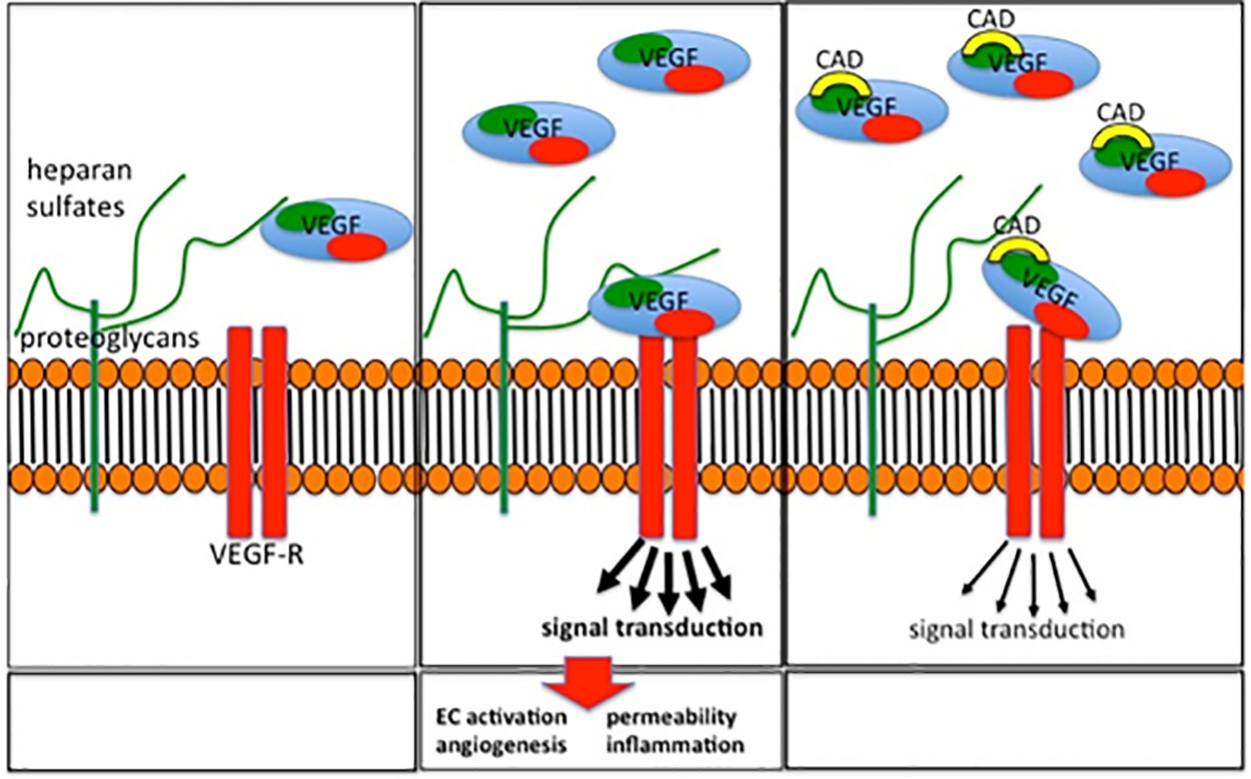
Postulated model of interactions between VEGF_165_, VEGFR-2, and CaD. VEGF_165_ binds to its co-receptor heparin sulfates (HS) of the endothelial glycocalyx with a specific binding site (left box) which stabilizes the VEGF-VEGF-R binding leading to phosphorylation of VEGFR-2 receptor, intracellular signaling and cell activation (middle box). CaD interacts with the heparin-binding domain of the VEGF_165_ (right box), thereby displacing HS from its binding site, and decreases VEGF-induced intracellular signaling. CaD also regulates VEGF_165_ activity by participating in the formation of unstable VEGF-VEGFR-2 complex.

We propose a CaD-induced mechanism of action involving VEGF_165_ inhibition (Fig 10). Cell surface HS regulates VEGF_165_ binding to VEGFR-2 and VEGF_165_-dependent phosphorylation of VEGFR-2 via binding to the heparin-like domain. In the presence of CaD, VEGF_165_, VEGFR-2 complex formation with cells surface heparan-sulfate proteoglycans is abrogated resulting into an unstable complex which is either fast degraded or VEGF_165_ binds to its receptors with low affinity. As a result, VEGF_165_-induced signaling processes, such as the phosphorylation of VEGFR-2, are decreased by CaD. It is also possible that in addition to binding to the ligand, CaD binds albeit with low significance to the HBD of VEGFR-2 partially blocking VEGF_165_ from interacting with its receptors and therefore, contributing to signaling inhibition.

Our proposed mechanism explains not only the CaD inhibitory effect on VEGF, but also the low rate of adverse effects as compared to VEGF antibodies in diabetes [27,58]. While VEGF antibodies completely block the effects of VEGF on the intracellular signaling pathways and thereby also block VEGF-induced signals which are necessary for endothelial cell survival, interference by CaD with the heparan sulfate binding sites reduces the binding of VEGF to its receptor and, therefore, reduces its effects on endothelial cells but does not abolish the effect of VEGF on its specific membrane-bound receptor, resulting in a modulatory effect on the VEGF overall action.

The severity of nephropathy is usually defined by proteinuria, which is closely correlated to renal damage. Following treatment with CaD, renal function was improved significantly as evidenced by decreased serum creatinine and urinary albumin. In addition, our experiments show that CaD prevents the increase and upregulation/activation of several pro-inflammatory cytokines (IL-6, CXCL1, IL-1ß, TNF-α, and MCP-1) that play a significant role in renal-disease progression. Although the anti-inflammatory properties of CaD have been previously reported [25,59,60], to the best of our knowledge, our report is the first involving a diabetic-nephropathy animal model. These findings are therefore important due to the pivotal role of inflammation in the pathogenesis of diabetic nephropathy.

CaD is reported to inhibit VEGF-induced endothelial permeability and protect against blood-brain-barrier leakage in diabetic mouse model. Such an effect was correlated with a decrease in the levels of VEGF in the retina [61], however, the decreased VEGF levels was reported to be due to a direct effect of CaD on VEGF expression [25]. Although we also observed down-regulation of VEGF production in the kidneys from STZ mice, we believe that this is a secondary phenomenon after improvement of endothelial cell function.

Interestingly, we observed a strong effect of CaD on diabetic neuropathy (DNP). Although not in similar settings, these results could be extrapolated to those of Sola-Adell et al., [61] who reported retinal neuroprotective effect of CaD in a diabetic mouse model. Moreover, Han et al., recently published promising efficacy data of CaD on DNP symptoms in human [62]. Further studies are however required to demonstrate the effectiveness of CaD in the treatment of peripheral DNP. Since CaD pharmacokinetics is already know [63], and it is currently used to treat vascular complications of diabetic retinopathy [64], our findings demonstrated the therapeutic potential of CaD in the early stages of DN given that at present, anti-VEGF antibody or tyrosine kinase inhibitors therapy for diabetic nephropathy is not warranted [65].

In summary, we demonstrated that CaD inhibits VEGF signaling and function in endothelial cells and that this effect is mediated via a novel mechanism interfering with the complex formation between VEGF, VEGF-R and HS. We could also show that CaD ameliorates diabetic nephropathy in a streptozotocin-induced diabetic mouse model by VEGF inhibition. We suggest a novel mechanism to interfere with VEGF signaling and suggest that the class of CaD compounds should be investigated further, particularly in the pathogenesis of diabetic nephropathy.

## Supporting information

S1 Fig Viability inhibition of CaD on HUVECs under normal culture conditions. HUVECs were exposed to CaD at the indicated concentrations and times, and viability measured by CCK-8 assay. Data are represented as percentage of untreated control from two independent experiments.
(TIF)

S2 Fig Effect of increasing concentrations of heparin on bt-VEGF_165_ binding to immobilized VEGR-1/2. Binding of biotinylated VEGF to VEGFR in the presence of heparin was investigated as described in materials and methods. Data are expressed as optical density (OD) at 450 nm from three independent experiments. *p < 0.05 vs. no heparin.
(TIF)
