# Correction: Calcium dobesilate reduces VEGF signaling by interfering with heparan sulfate binding site and protects from vascular complications in diabetic mice

**DOI:** 10.1371/journal.pone.0244353

**Published:** 2020-12-16

**Authors:** Florence Njau, Nelli Shushakova, Heiko Schenk, Vera Christine Wulfmeyer, Robin Bollin, Jan Menne, Hermann Haller

The following information is missing from the Funding statement: This work was also supported by a grant from the Else-Kröner-Fresenius Foundation to HA: Projekt 2017_A96.
