# Supplementary figures and images for: Calcium dobesilate reduces VEGF signaling by interfering with heparan sulfate binding site and protects from vascular complications in diabetic mice
Source: PLoS One. 2020 Jan 14;15(1):e0218494. doi: 10.1371/journal.pone.0218494 (PMC6959593; doi:10.1371/journal.pone.0218494)

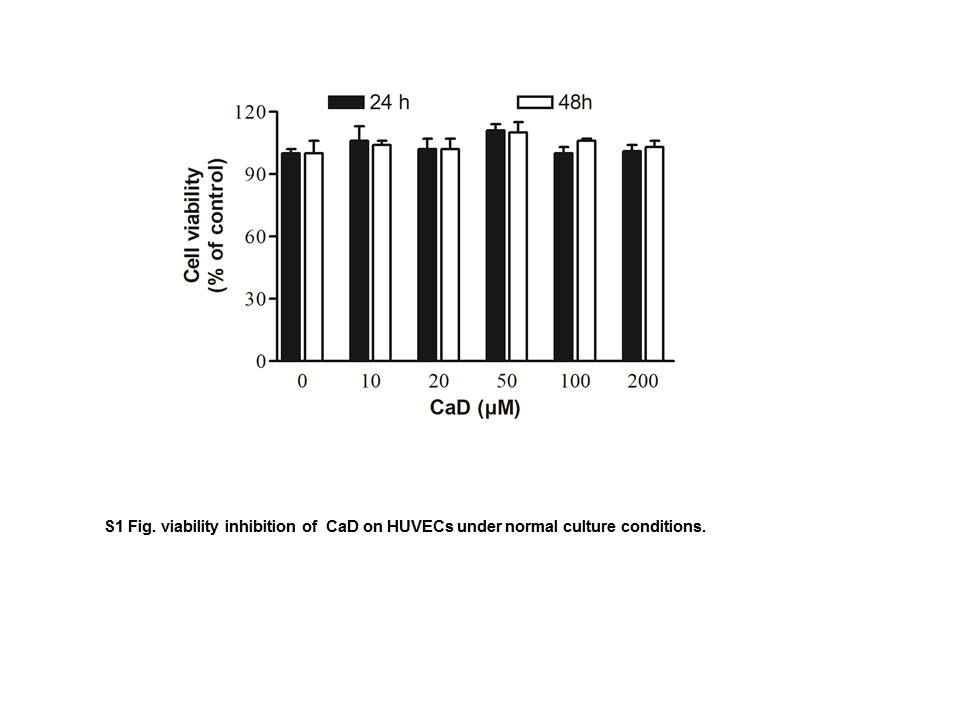

Supplement: S1 Fig — HUVECs were exposed to CaD at the indicated concentrations and times, and viability measured by CCK-8 assay. Data are represented as percentage of untreated control from two independent experiments. (TIF) [file pone.0218494.s001.tif]

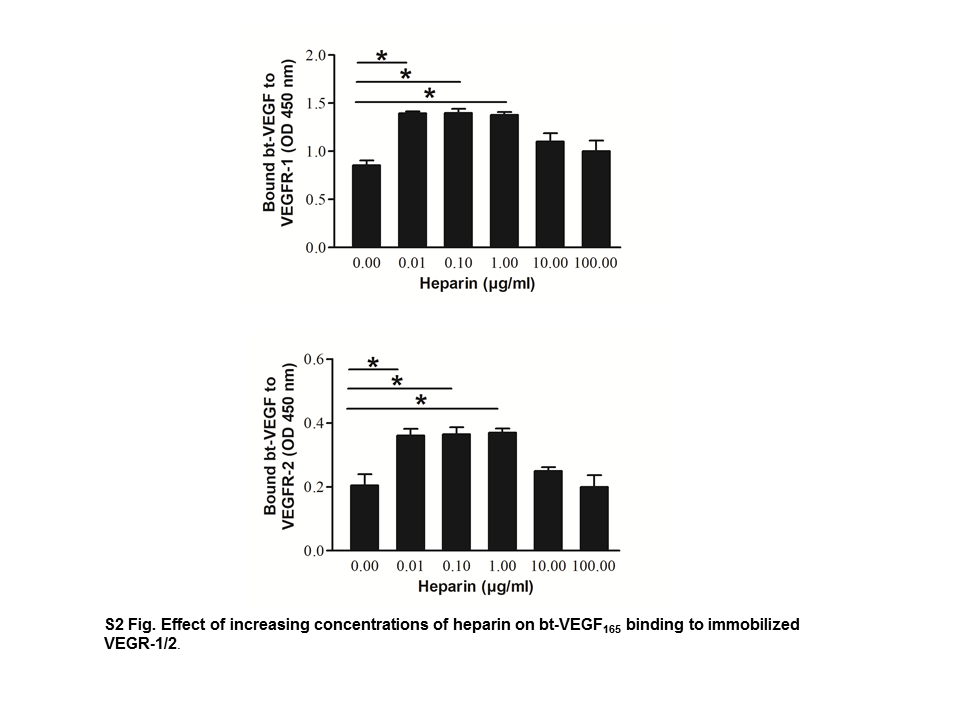

Supplement: S2 Fig — Binding of biotinylated VEGF to VEGFR in the presence of heparin was investigated as described in materials and methods. Data are expressed as optical density (OD) at 450 nm from three independent experiments. *p < 0.05 vs. no heparin. (TIF) [file pone.0218494.s002.tif]
